# Evaluating the Rheological Properties of High-Modulus Asphalt Binders Modified with Rubber Polymer Composite Modifier

**DOI:** 10.3390/ma14247727

**Published:** 2021-12-14

**Authors:** Xiaorui Zhang, Chao Han, Jun Yang, Xinquan Xu, Fan Zhang

**Affiliations:** 1 School of Transportation, Southeast University, Nanjing 211189, China; zxr@seu.edu.cn (X.Z.); hc1527@jsti.com (C.H.); 2 Guangdong Hualu Transportation Technology Co., Ltd., Guangzhou 510420, China; xuxinquan@seu.edu.cn

**Keywords:** high-modulus asphalt binder, rubber polymer composite modifier (RPCM), rheological properties, fluorescence microscope

## Abstract

With the increasing traffic loading and changing climatic conditions, there is a need to use novel superior performing pavement materials such as high-modulus asphalt binders and asphalt mixtures to mitigate field distress such as rutting, cracking, etc. This laboratory study was thus conducted to explore and substantiate the usage of Rubber Polymer Composite Modifier (RPCM) for high-modulus asphalt binder modification. The base asphalt binder used in the study comprised A-70# Petroleum asphalt binder with RPCM dosages of 0.25%, 0.30%, 0.35%, 0.40% and 0.45%, separately. The laboratory tests conducted for characterizing the asphalt binder rheological and morphological properties included the dynamic mechanical analysis (DM), temperature-frequency sweep in the dynamic shear rheometer (DSR) device, bending beam rheometer (BBR), and florescence microscopic (FM) imaging. The corresponding test results exhibited satisfactory compatibility and potential for using RPCM as a high-modulus asphalt binder modifier to enhance the base asphalt binder’s rheological properties, both with respect to high- and low-temperature performance improvements. For the A-70# Petroleum asphalt binder that was evaluated, the optimum RPCM dosage was found to be 0.30–0.35%. In comparison to styrene–butadiene–styrene (SBS), asphalt binder modification with RPCM exhibited superior high-temperature rutting resistance properties (as measured in terms of the complex modulus and phase angle) and vice versa for the low-temperature cracking properties. Overall, the study beneficially contributes to the literature through provision of a reference datum toward the exploratory usage of RPCM for high-modulus asphalt binder modification and performance enhancements.

## 1. Introduction

The continuous economic development and rapid evolution of vehicular transportation as the main means of human travel and goods movement have led to increased traffic volume and heavier truck axle loads on the road network. Coupled with insufficient maintenance, resource challenges, and climate change, this has undesirably led to the induction of widespread distresses, such as rutting, cracking, pot-holing, raveling, etc., on asphalt pavements [1]. With this increased traffic loading and changing climatic (environmental) conditions, traditional asphalt mixtures may no longer be able to meet the current pavement design specifications and requirements against such heavy traffic loading, particularly with respect to the truck axle weights [2]. Therefore, consideration of the local climate and site-specific traffic loading conditions has become a critical design input for asphalt mixtures [3].

Rutting is one of the main causes of early damage to asphalt pavements in the high-temperature areas of China [4]. During the asphalt mixture design stage in the laboratory, rutting is mainly mitigated through aggregate gradation optimization, asphalt binder modification, adding admixtures, and volumetric optimization such as decreasing the asphalt binder content with the primary goal of increasing the stiffness (modulus) of the asphalt mixture. Use of high-modulus asphalt binders is also another means to produce stiff mixtures with superior rutting resistance performance [5].

If good bonding can be achieved with the aggregates, high-modulus asphalt binders can effectively improve the overall bearing capacity of semi-rigid or flexible pavements under heavy traffic conditions [6]. Some of the methods used to improve the modulus of asphalt binders, alike, include: (a) using low-grade hard asphalt binders, such as 30# or 50# asphalt binders; (b) adding polymers into the asphalt binders or asphalt mixtures for modification, such as styrene–butadiene–styrene (SBS), polyethylene (PE), ethylene–vinyl acetate (EVA), and various high-modulus agents (i.e., Polymer Rubber(PR) PLAST S and PR MODULE), etc [7].

SBS modifier is one of the most widely used technologies during asphalt modification. Xiao et al. [8] found that high-modulus agents could enhance the rutting resistance of styrene–butadiene–styrene (SBS)-modified asphalt binders. Their [8] findings indicated that asphalt binders modified with high-modulus agents had better fatigue performance than the asphalt binders modified with anti-rutting agents at various stress levels. Rad Spunrie is cheaper, but its performance is unstable, especially in terms of the dynamic modulus and low-temperature performance [9]. Geng et al. [10] found that high-modulus asphalt binders (HMAB) had higher stiffness and elasticity, but lower thermal cracking resistance than the neat asphalt binders. Zou et al. [11] suggested that the contents of two high-modulus modifiers (namely PR PLAST S and PR PLAST Module) should be 10% by weight of the asphalt binder in terms of the high-temperature performance and fatigue properties. Wang et al. [12] investigated the influence of the Trinidad Lake Asphalt (TLA) dosages on high- and low-temperature performances, moisture resistance, and fatigue resistance of HMAC modified with TLA and polyester fiber. The results successfully revealed the composite modified mechanism of the TLA and polyester fiber and suggested that the best volumetric combination for a high-modulus modifier was 30% TLA + 3‰ polyester fiber. Khiavi et al. [13] used a hard-penetration bitumen (HPB) to prepare high-modulus asphalt mixtures (HMAM). The results revealed that HMAM (i.e., Enrobés à Module Élevé Class 2 (EME2) and Grave Bitumen Class 5 (GB5)) with higher asphalt binder content (i.e., 6% by mass) of the HPB had better rutting and fatigue resistances than the control mixtures. It was also observed that HPB could improve the moisture sensitivity of HMAM by 22%.

These high-modulus modifiers have some drawbacks, such as long mixing time, large particle size, and poor storage stability. Some high-modulus modifiers also need higher mixing and compacting temperatures, which makes it inconvenient to use in low-temperature seasons. In addition, it is associated with challenges such as high energy consumption, high pollution, insufficient storage stability, and easy aging [14]. Thus, the application of high-modulus agents in the asphalt binder modification process helps to mitigate against rutting, improving fatigue resistance, and extending the service life of asphalt pavements [15].

In their study, Zou et al. [16] found that the particle size of high-modulus modifiers and the relative viscosity of the modified asphalt binder increased as a function of time when measured using the scanning electron microscope (SEM) and fluorescence microscope (FM) tests, respectively. The SEM and FM results indicated that the network structures formed by high-modulus modifiers in the asphalt binder could potentially improve the high temperature and moisture performance of the asphalt binder. Scanning electron microscope (SEM) images showed that the modified regeneration method could improve the bonding and interface structures of modified asphalt binder [15]. Wang et al. [17] studied the rheological characteristics of high-modulus asphalt binders modified with rock asphalt (RA) and PR using temperature-frequency sweep, multiple-stress creep recovery (MSCR), and linear amplitude sweep (LAS) tests. As theoretically expected, the two modifiers exhibited potential to improve the high-temperature performance of the asphalt binders, but with a decrease in fatigue resistance. By comparison, RA had little influence on the fatigue performance of the modified asphalt binder.

Based on the above background and review of the literature, the objective of this study was to investigate the effects of different dosages of RPCM (Rubber Polymer Composite Modifier) on the rheological properties and morphology of high-modulus asphalt binders. Five different dosages of high-modulus modifiers, which mean 0.25%, 0.30%, 0.35%, 0.40%, and 0.45% (by weight of the HMAC), were added to A-70# petroleum asphalt binders to produce the HMABs. Temperature and frequency sweep tests were conducted to investigate the high-temperature performance and viscoelastic characteristics of HMAB using the Dynamic Shear Rheometer (DSR) test device. Bending beam rheometer (BBR) tests were also conducted to investigate the low-temperature performance of the HMAB. Overall, the study goal and research methodology were tailored toward solving the challenges associated with the use of high-modulus modifiers and promote the application of high-modulus asphalt mixtures in environment-friendly and long-life pavements.

In the subsequent section of the paper, the materials and laboratory test methods are discussed, followed by a presentation of the test results and analysis thereof. A morphological analysis of the HMAB through fluorescent imaging was then presented, and thereafter, the paper concludes with a summary of key findings and recommendations.

## 2. Materials and Laboratory Test Methods

The materials used in the study as well as the laboratory tests conducted are discussed in this section. This includes the raw materials, test methods, and preparation of HMAB.

### 2.1. Raw Materials

The raw materials used in this study comprised A-70# Petroleum asphalt binder and RPCM materials. These raw materials are discussed in the subsequent text.

#### 2.1.1. Asphalt binder

The asphalt binder used was #70 A-grade Petroleum that was supplied by Jiangsu Baoli Asphalt Co., Ltd. Of China. Its technical indices are listed in Table 1 and satisfactorily meet the Chinese JTG F40-2004 specification requirements for asphalt pavement construction [18].

The asphalt binder performance grading (PG) system based on the rheological properties of asphalt was introduced in the United States of America, as a product output of the Strategic Highway Research Program (SHRP) [19,20]. To evaluate the high-temperature performance of A-70# Petroleum asphalt binder, DSR test device was used to grade asphalt binder at high temperature. Based on G*/Sin δ ≥ 1.0 kPa in the unaged condition and G*/Sin δ ≥ 2.2 kPa after RTFOT aging, the high-temperature performance grade of A-70# Petroleum asphalt binder was determined to be PG 64-28.

#### 2.1.2. RPCM Material

RPCM is a high-modulus modifier, which comprises waste rubber and plastic high polymer as the base material, combined with hyperdispersant, polar interface compatibilizer, anti-aging agent, and other compounds. After mixing and granulating with a special extrusion process, it can reach the micron scale level and disperse evenly in the asphalt mortar at 180 °C temperature through 45 min. Under the action of the dispersant compound, the high polymer in modifier can form a network structure with high stability and strength with the molecular chain to improve the strength and modulus of the asphalt binder by limiting the movement of the small molecular chains of the asphalt binder. As a result, the modifier can greatly improve the modulus and high-temperature performance of the corresponding asphalt mixture. The technical indices of RPCM are listed in Table 2.

According to the GB/T 3682 specification [21], RPCM satisfactorily meets the requirement that the melt mass flow rate (MFR) of thermoplastic should be greater than 1.0 g/10 min.

### 2.2. Test Methods and Sample Preparation

The test methods are discussed in the subsequent text and includes the following: dynamic mechanical analysis, temperature-frequency sweep, BBR, and florescence microscope. HMAB preparation and test matrix are also discussed herein.

#### 2.2.1. Dynamic Mechanical Analysis

Dynamic Mechanical Analysis (DMA) is a technique used to study the viscoelastic behavior of materials. It is most useful for studying the mechanical behavior of viscoelastic materials under cyclic stress (or strain). DMA can highlight the rheological properties of an asphalt binder by measuring the dynamic mechanical properties of asphalt binder in a certain range of temperature and frequency. The temperature and frequency sweep tests were conducted using the DSR device to evaluate the viscoelastic properties of the asphalt binder.

**Figure 1:**
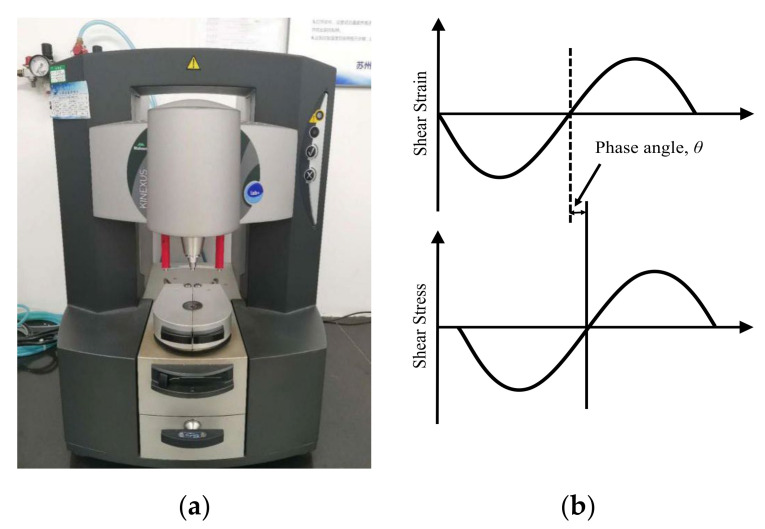
(**a**) Kinexus DSR device and (**b**) oscillation mode and operating principle.

The Kinexus ultra+ rotational rheometer produced by Malvern Instruments was used and is shown in Figure 1. The torque range is 3 nN∙m~250 mN∙m. The frequency range is 1 μHz~150 Hz. Its temperature control range is −30 °C~200 °C. The Kinexus rheometer has a large frequency and temperature range, which can be used to study the viscoelastic properties of asphalt binders and asphalt mixtures over large temperature and frequency ranges, respectively.

The DSR test uses a thin asphalt binder sample sandwiched between two metal circular plates with 8 mm diameter. The lower plate is fixed, while the upper plate oscillates back and forth across the sample at a constant angular velocity to create a shearing action under the control of a motor. The shear force on the specimen is a sinusoidal stress with an angular velocity of ω. Under stable periodic oscillation, the stress on the specimen is a sinusoidal function of the angular velocity.

When the shear stress oscillation is small, the specimen shows linear viscoelastic behavior—that is, the shear strain of the specimen shows the same oscillating state. The phase angle of the asphalt binder (δ) represents the time lag between the applied shear stress and the resulting shear strain. The complex shear modulus (G*) is the amplitude ratio of the applied shear stress to the resulting shear strain.

*(1)*  *The Temperature Sweep Test (TST)*

If the sweeping temperature of the asphalt binder is carried out at a low temperature, the control strain must be set in the linear viscoelasticity range of the asphalt binder. This is because the linear viscoelasticity range of the asphalt binder is too small at low temperatures. However, too small a control strain will affect the accuracy of the test data, resulting in large differences, poor stability, and poor reproducibility of test results. To ensure quality and validity of the test data, the sweeping temperature range was set to 58 °C~106 °C in this study.

In the temperature sweeping test, the diameter of the plates was 25 mm, and the gap between the rotating plate and the fixed plate was 1.0 mm. The magnitude of the loading strain was controlled to 0.1%. The loading frequency was 1.592 Hz (10 rad × s^−1^). The temperature range in the test was 58~106 °C, with a sweeping temperature interval of 6 °C.

*(2)*  *The Frequency Sweep Test (FST)*

The frequency of the wheel load and the actual load induced on the pavement is partly related to the driving speed and wheel size of the vehicle. The relationship between the driving speed and wheel size on the pavement is as follows:

(1) $$T=\frac{C}{v}$$

(2) $$\omega =\frac{2\pi }{T}$$

(3) $$f=\frac{1}{T}$$

where *C* = wheel circumference (m);

*v =* vehicle speed (m/s);

*T =* load cycles/load (s);

*f =* loading frequency (Hz).

To evaluate and quantify the fatigue performance of the asphalt binder, test temperatures of 5, 20, and 35 °C, separately, were selected for the frequency sweeping test to obtain the complex modulus master curve [22] of asphalt binder samples. The frequency range of the FST was 0.1~100 rad × s^−1^. The complex modulus of the asphalt binder sample correspond to the test results with low frequency when the temperature is higher than 20 °C and vice versa when the temperature is lower than 20 °C, respectively. Since the test temperature range was between 5 °C~35 °C, 8 mm plates with a 2 mm gap were selected and used for the test. The control strain was set at 0.1%. The loading frequency range was 0.1~100 rad × s^−1^.

*(3)*  *Asphalt binder Sample Preparation*

In the TST, a mold with a diameter of 25 mm was selected and utilized to prepare the asphalt binder sample to avoid the separation of the upper and lower plates due to the small sample size at low temperatures. A mold with a diameter of 8 mm was selected and used to prepare the asphalt binder sample in the FST.

To ensure stable performance for the asphalt binder sample during the tests, the asphalt binder was stirred evenly with no bubbles inside during preparation of the sample. A mold, matching the DSR device, was used for pouring the sample. The mold and the sample, were thereafter, placed into a refrigerator for at least 2 h to cool and solidify the hot liquidus asphalt binder. Within five minutes of removal from the refrigerator, the sample was demolded and quickly packed in a clean sample bag for storage to 20 °C (ambient temperature) for at least 4 h prior to testing.

#### 2.2.2. The Bending Beam Rheometer (BBR) Test

At test temperatures below 0 °C, the DSR test can be used to evaluate the low-temperature performance of asphalt binders. However, at such low temperatures, the linear viscoelastic range of the sample is too small to be controlled, often leading to instability in the test results. On this basis, the BBR test was selected to evaluate the low-temperature properties and performance of the asphalt binder.

The standard size of the asphalt binder beam for BBR testing is 127 mm long by 12.7 mm thickness by 6.35 mm width. The automated BBR test loading process is divided into the following four stages: (a) apply an initial load of 980 mN within 1 s; (b) reduce the load to 35 mN and maintain it for 20 s; (c) apply a test load of 980 mN and maintain it for 240 s; and (4) reduce the load back to the contact load of 35 mN and remove the sample, thus signifying BBR test completion. In this process, a computer automatically records and calculates the load and deformation values from 0.5 s at an interval of 0.5 s. For sample preparation, mold matching the BBR test device were used for preparing the BBR beam samples.

### 2.3. Fluorescence Microscope (FM)

Fluorescence microscopy is an important tool for investigating the dispersion of rejuvenators in the aged asphalt binder phase. A drop of the asphalt binder sample was placed between two glass slides. Thereafter, it was heated at 163 °C and covered with the slide for 10 min. A uniform asphalt binder sample film was formed to be tested. The sample was observed in a fluorescence microscope with a fluorescent filter cube turret above the objective lenses. Photo micrographs were obtained with a digital camera and thereafter, magnified 4 × 100 times.

### 2.4. HMAB Preparation

The HMAB sample preparation and test matrix are discussed in this subsection. The text discussions also include the volumetric proportions and percentage dosages of the modifier (RPCM) into the asphalt binder.

To study the effects of RPCM on the asphalt binder (AB), HMAB was prepared using a high-speed shear homogenizer in the laboratory. The sample preparation process adhered to following four steps:

Step 1: The asphalt binder was melted into a liquid state in a constant temperature oven at 130~140 °C for about 1 h;

Step 2: The RPCM was slowly added into the asphalt binder at 130~140 °C, and then mixed using a high-speed shear homogenizer at 2500~3500 r/min for 10~15 min;

Step 3: The asphalt binder and RPCM were mixed rapidly at a speed of 5000–6000 r/min for 45 min;

Step 4: The asphalt binder and RPCM blend was then put in a 170 °C constant temperature oven to allow swelling development for about 3 h. After mixing, the sample was formed and placed into an environmental curing box for about 2 h in readiness for testing.

Five types of high-modulus modified asphalt binders (namely 70#-0.25%, 70#-0.30%, 70#-0.35%, 70#-0.40%, and 70#-0.45%, respectively) were produced according to Steps 1~4 A-70# Petroleum asphalt binder and SBS-modified asphalt binder were heated and liquidified without adding RPCM. These were stirred using a high-speed shear homogenizer for 60 min, kept in a 170 °C constant temperature oven for 3 h, and then formed into samples to simulate aging during the preparation of high-modulus modified asphalt binder.

## 3. Rheological Test Results, Analysis, and Synthesis

The test results are presented and discussed below. These test results include temperature sweep, frequency sweep, BBR, and morphological analysis.

### 3.1. The Temperature Sweep Test Results

Temperature sweep tests were carried out on seven different types of asphalt binder samples. The output data included the complex modulus (G*), rutting parameter (G*/Sinδ), phase angle (δ), and temperature.

#### 3.1.1. Complex Modulus of HMAB

**Figure 2:**
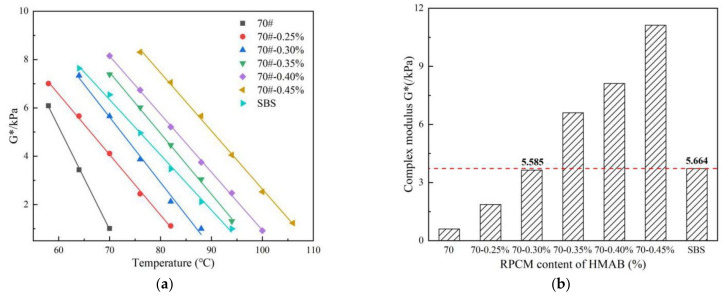
G*-temperature results at: (**a**) different RPCM contents, and, (**b**) 70 °C.

The relationship between the complex modulus (G*) and temperature for the seven asphalt binders evaluated as plotted in Figure 2.

As shown in Figure 2a, the complex modulus decreases exponentially with an increase in temperature. Adding RPCM into the base asphalt binder can potentially improve the high-temperature strength and stiffness of the asphalt binder [23]. However, the deformation resistance of the asphalt binder will still gradually decrease at higher temperatures. As shown in Figure 2b, the complex modulus of HMAB with different RPCM contents is 3.2, 6.1, 11.0, 14.2, and 18.6 times that of the base asphalt binder, respectively, when the experimental temperature is around 70 °C. That is, as theoretically expected, the stiffness is increasing with an increase in the modifier (RPCM) dosage [24].

At high temperature, it was observed that the complex modulus of HMAB increased nonlinearly with an increase in the RPCM content. The growth rate of the complex modulus was also observed to be slower when the RPCM content was less than 0.35%, meaning that RPCM has the potential to reduce the temperature sensitivity of the asphalt binder. Figure 2 also shows when the RPCM content is more than 0.30%, the complex modulus of HMAB is higher than that of the SBS-modified asphalt binder. Thus, to ensure effective modulus improvement on the base asphalt binder, it is suggested that the content of RPCM in A-70# Petroleum asphalt binder should not be less than 0.30%.

#### 3.1.2. Phase Angle of HMAB

**Figure 3:**
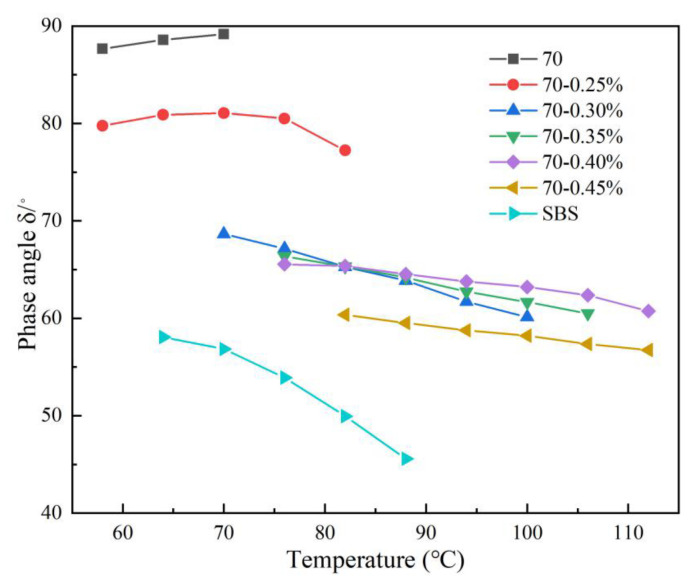
δ-temperature results as a function of temperature and RPCM dosage.

Phase angle (δ), which represents the material performance in terms of the viscous or elastic behavior, was experimentally determined by measuring the time lag between applied sinusoidal stress and the corresponding strain response [25]. The relationship between the phase angle (δ) and temperature for the seven evaluated asphalt binders (i.e., HMAB samples) are graphically illustrated in Figure 3.

Phase angle can reflect the elastic and viscous properties of asphaltic materials. The larger the δ value is, such as 90°, the stronger the viscous behavior, and the weaker the elastic property for the asphalt binder. In Figure 3, with an increase in the content of high-modulus (RPCM) modifier, the phase angle of HMAB shows a general decreasing trend, indicating that the elastic properties of the asphalt binder under high-temperature regimes can be improved by adding RPCM. Thus, the addition of the RPCM can potentially bridge the gap between the base asphalt binder and SBS-modified asphalt binder to some extent.

For viscoelastic materials, temperature and phase angle are typically inversely correlated (i.e., reducing the temperature increases the phase angle and vice versa). For the base asphalt binder in Figure 3, however, the phase angle exhibited an increasing trend as the temperature was increased, with no stable region during the total range of temperature sweep measurements. Asphalt, which shows elasticity at low temperature and viscosity at high temperature, is the typical viscoelastic material. In the dynamic shear test, with the increase of temperature from lower to higher, asphalt material becomes soft. As the result, the viscosity component is enhanced, and the elastic component is reduced, such that the phase Angle of asphalt increases with the increase of temperature. Within the temperature range of 60 to 70 °C, the 0.25% RPCM dosage shows an almost similar phase-angle response trend as the base asphalt binder. For temperatures over 70 °C, the 0.25% RPCM dosage shows a similar phase-angle response trend as the SBS-modified asphalt binder because of the same component of the polymer phase at these high temperatures.

Compared to the base asphalt binder, the SBS-modified asphalt binder shows lower phase angles. This means that the SBS-modified asphalt binder has better recovery ability than the base asphalt binder nor the RCPM modified asphalt binder. For the RPCM modification, in Figure 3, a significant difference in the phase-angle response trends with increasing RPCM content mainly occurs in the temperature range of 70 to 90 °C. This indicates that the structures formed by RPCM in the base asphalt binder dominate the rheological behavior of HMAB and retard or even reverse the growth trends of the phase angle with increasing temperature by increasing the elastic response of HMAB.

#### 3.1.3. Rutting Factor of HMAB

**Figure 4:**
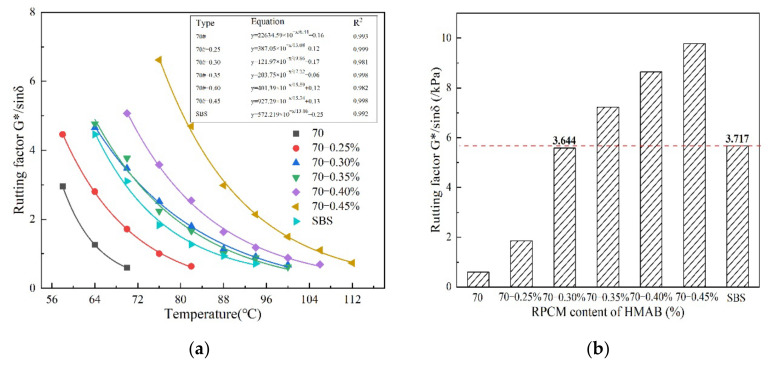
G*/Sinδ temperature results at: (**a**) different RPCM contents, and, (**b**) 70 °C.

The rutting factor, G*/sinδ, reflects the ability of the asphalt binder to resist deformation, which inherently decreases with an increase in temperature [23,24,25]. In general, asphalt binders with a high rutting factor in magnitude are deemed to have more potential to resist deformation and rutting at high temperatures. The G*/Sinδ test results for the seven asphalt binders evaluated in this study are graphically shown in Figure 4 as a function of temperature.

With an increase in the RPCM content, the rutting factors correspondingly increased in magnitude, thus indicating an improvement in the rutting resistance of the composite high-modulus modified asphalt binder, namely HMAB. The quantitative increase in the magnitude of the rutting factors, as a function of RPCM contents, is similar to the complex modulus, which also shows a nonlinear relationship. In the SHRP specification [19,20,26], the value of G*/Sinδ must be more than 1.0 kPa for asphalt binders to mitigate the rutting phenomenon. As a result, the rutting factor for which G*/Sinδ > 1.0 kPa for the base asphalt binder in Figure 4 should not exceed 64 and 84 °C for the SBS-modified asphalt binder. For HMABs with 0.25%, 0.30%, 0.35%, 0.40%, and 0.45% RPCM content, the temperature benchmarks are 64, 76, 80, 88 and 100 °C, respectively. This again illutrates the potential of the RPCM to reduce the temperature sensitive of the asphalt binder and enhance its high-temperature rutting resistance potential.

Figure 4b shows a barchart relationship between the rutting factor and RPCM contents of HMAB at 70 °C. From the figure, the rutting factor exhibits a nonlinear increasing with an increase in the RPCM contents at high temperatures. The rutting factors at 0.3% and 0.35% RPCM dosages are 11.2 and 15.1 times that of the base asphalt binder, respectively. Compared to the rutting factors for the SBS-modified asphalt binder, these results suggests that the RPCM content in base asphalt binder should be more than 0.30%—as to achieve enhanced performance that is above the SBS modification.

### 3.2. The Frequency Sweep Test Results

A series of frequency sweep tests were performed on the asphalt binder samples to generate master curves of the complex modulus and phase angle, respectively. All the asphalt binder samples were tested in the frequency range of 0.1~100 rad × s^−1^ at 5, 20, and 35 °C. Ten points for each frequency magnitude, giving a total of 30 frequency points, were selected to check and quantify the extension of the linear viscoelastic domain.

#### 3.2.1. Translational Shift Factors for HMAB

**Figure 5:**
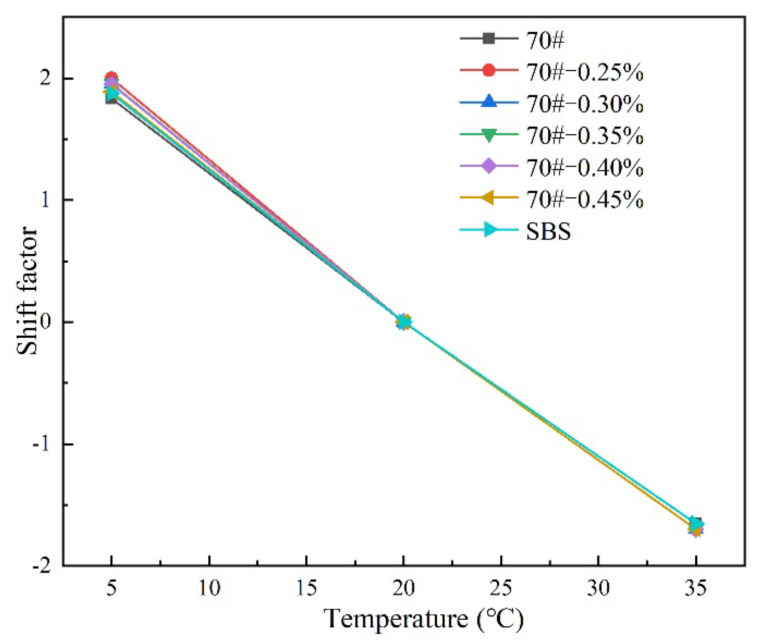
Shift factors for HMAB under different temperatures.

The translation shift factors of all asphalt binder samples were obtained at different temperatures through translation to a reference temperature. The results of this analysis are graphed in Figure 5, with 20 °C used as the reference temperature.

As shown in Figure 5, the shift factors are similar, with the graphical curves overlapping each other particular in the high temperature domain. The shift factors averaged about −1.653, 0.000, and 2.000 at 35, 20 (reference temperature), and 5 °C, respectively. The quality of asphalt binder is partly determined by the crude oil and production process. More specifically, the shift factor of the base asphalt binder is more dependent on the crude oil rather than the production process. Therefore, the overlapping curves in Figure 5 partially substantiate the consistency and quality of the tested asphalt binder materials, which also suggest a homogeneous blend during the RPCM modification process.

#### 3.2.2. Master curves of the Complex Modulus

**Figure 6:**
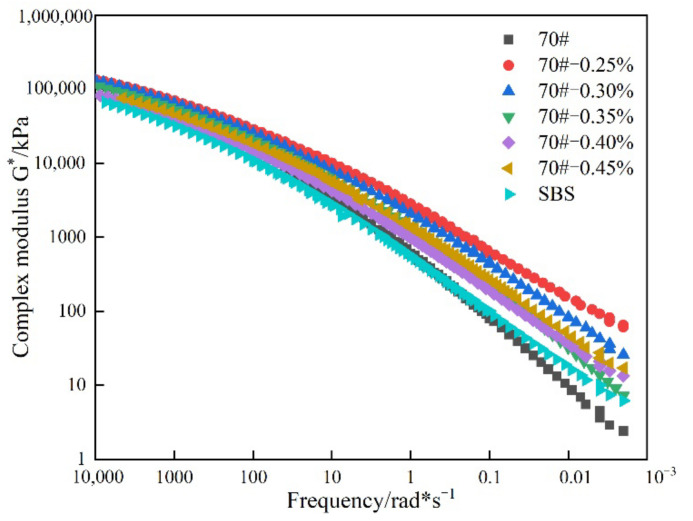
Master curves for the complex modulus of the asphalt binders.

Based on the Williams–Landel–Ferry (WLF) superposition principle [27], the master curves of asphalt binder complex modulus were constructed at a reference temperature of 20 °C, a temperature which corresponds to near ambient conditions. The generated master curves are plotted in Figure 6.

The viscoelastic behavior of asphaltic materials has a clear dependence on temperature and loading time. According to the principle of time-temperature equivalence for viscoelastic materials such as asphalt binders, the high-temperature state corresponds to longer loading time and vice versa for the low-temperature state, i.e., corresponds to shorter loading time. For simplicity of analysis in this paper, the temperature and loadng time effects were theoretically assumed to be equivalent.

As can be seen from Figure 6, the complex modulus of the base asphalt binder after adding RPCM was significantly improved, particularly in the low frequency domain corresponding to longer loading times and high temperatures. The base asphalt binder yielded the least complex modulus in the low frequency domain while the 0.25% RPCM-HMAB had the highest stiffness at almost all the tested frequencies. Compared to the base asphalt binder, the SBS-modified asphalt binder exhibits relatively lower moduli values at higher loading frequencies and vice versa at lower loading frequencies, respectively.

When the loading frequency is 10,000~100 rad × s^−1^, Figure 6 shows that the complex modulus did not change significant as a function of the RPCM dosage, with the curves nearly overlapping each other. However, when the loading frequency is 100~0.001 rad × s^−1^, the complex modulus exhibited an increasing trend with an increase in the RPCM content, with the curves visually distinct particularly within the 0.01~0.001 rad × s^−1^ frequency domain. However, the highest and lowest moduli values are indicated for 0.25% and 0.35% RPCM, respectively, with the second highest being 0.30% RPCM. In fact, 0.30% RPCM dosage exhited greater moduli values than the higher dosages (i.e., 0.35%, 40%, and 0.45%, respectively) at all the loading frequencies evaluated. This could suggest that 0.30% is the optimum RPCM dosage. Nonetheless, the reasons for 0.25% being higher in terms of the moduli magnitude are unknown, but could be related to human test/analysis errors and warrants further research in future follow-up studies.

Based on the master curves in Figure 6, it is apparent that RPCM modification has more profound impacts in the lower loading frequency domain corresponding to high temperatures when the viscoelastic asphalt binder is more prone to rutting. This is a desirable phenomenon to mitigate against rutting since asphalt binders are more susceptible to deformation at high temperatures and longer loading times. That is as the complex modulus decreases at high temperatures, the asphalt binder becomes less stiff and thus, able to easily deform without resisting large stresses under longer loading times, which is an undesirable phenomenon that, among others, can be minimized through RPCM additive as demonstrated in Figure 6.

#### 3.2.3. Master curves of the Phase Angle

**Figure 7:**
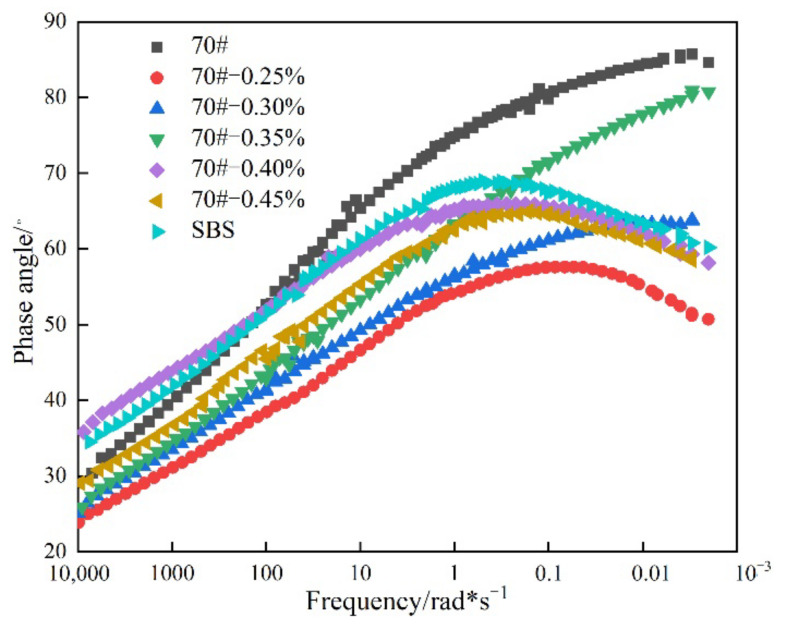
Master curves for the phase angle of the asphalt binders.

Based on the shift factors in Figure 5, master curves for the phase angle were generated at a reference temperature of 20 °C that simulated near ambient temperature conditions. These master curves are graphically shown in Figure 7.

As evident in Figure 7, the phase angle decreased significantly with an increase in both the RPCM content and loading frequency (i.e, shorter loading time). This means that the elasticity of the asphalt binder significantly improved. The base asphalt binder shows the highest phase angle (in magnitude) at the lower frequency range (10^−3^~10^2^ rad×s^−1^), which continuously increased as the temperature decreased, with no plateau within the whole range of the frequency sweep.

In contrast to the base asphalt binder and with the exception of 0.45% RPCM, the phase angle for the RPCM-modified asphalt binders appear to have hyperbolic shapes with peaks occurring within the frequency range of 0.10 to 1.00 rad × s^−1^. Likewise, the SBS-modified asphalt binder exhibited a similar response trend with a peak occurring within the frequency range of 0.10 to 1.00 rad × s^−1^, albeit that its phase angle is for the most part greater than the RPCM-modified asphalt binder, i.e., more viscous. In general, asphalt binders with low phase angles tend to be more elastic, with desirably enhanced resistance to, and vice versa, for high phase angle asphalt binders, i.e., more viscous.

### 3.3. The BBR Test Results

**Figure 8:**
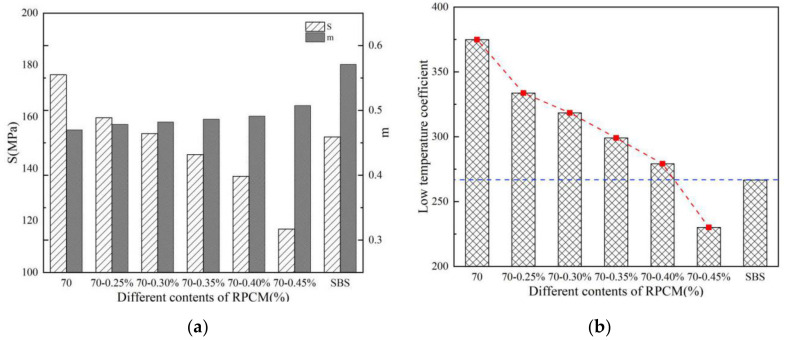
(**a**) Creep stiffness; (S) m value; (**b**) low-temperature coefficient.

The effects of the RPCM dosage on the stiffness (S) and creep rate (m value) of the asphalt binders were evaluated using the BBR test. Creep stiffness modulus S is the ratio obtained by dividing the measured maximum stress by the measured maximum bending strain. Creep rate m is the absolute value of the slope of the logarithm of the stiffness curve versus the logarithm of time. The BBR test temperature was −10 °C. The corresponding barchart results are shown in Figure 8.

For grading of the low-temperature performance of asphalt binders, the PG system limits the creep stiffness modulus to 300 MPa, i.e., S ≤ 300 MPa, and the creep rate to a minimum of 0.30, i.e., m value ≥ 0.3, at 60 s. As shown in Figure 8, with an increase in the RPCM dosage, the creep stiffness modulus (S) and low-temperature coefficient (λ) gradually decreased, which is significant when the RPCM content is greater than 0.35%. For viscoelastic materials, the larger the stiffness of the asphalt binder, the more brittle it is, and the more susceptibility it is to low-temperature cracking. Thus, the declining graphical trends with increasing RPCM dosage in Figure 8 suggest improvement in resistance to low-temperature cracking.

From Figure 8, it is also visually noted that the creep rate (i.e., m value) gradually increased, which is also significant when the RPCM content is greater than 0.35%. This indicates that when the temperature decreases and the RPCM content increases, the response of the asphalt binder is consistent with the response phenomenon of viscoelastic materials whose stiffness gradually decreases, with the potential for enhanced resistance against low-temperature cracking. However, the SBS-modified asphalt binder with the largest creep rate (i.e., m value) in Figure 8 indicates better ductility and stress relaxation properties, with potential for theoretically higher resistance to cracking in low-temperature environments than the RPCM-modified asphalt binders.

Compared to the other asphalt binders in Figure 8b, 0.45% RPCM yielded the smallest low-temperature coefficient (λ) in magnitude. Based on this parameter (λ), this theoretically infers that 0.45% RPCM had the best low-temperature performance, with the base asphalt binder being the poorest. In general, a larger S/m indicates a quicker growth rate for the creep stiffness (S), which may undesirably mean severe erosion of the base asphalt binder.

## 4. Morphological Test Results, Analysis, and Synthesis

**Figure 9:**
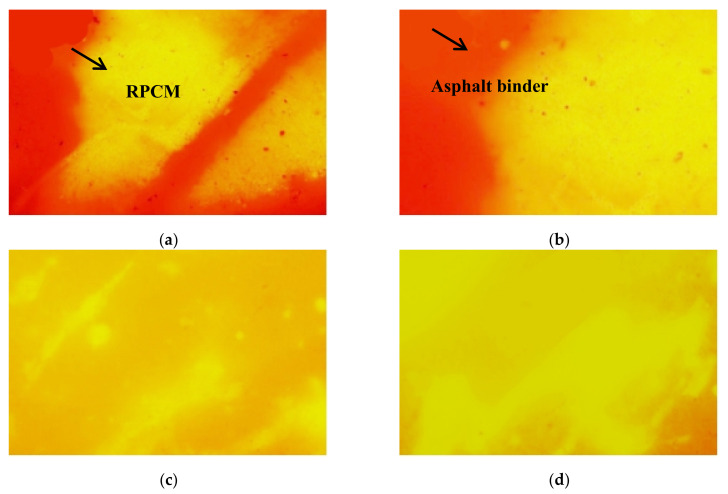
Fluorescence micrograph of 0.35% RPCM-modified HMAB under different evolution times: (**a**) 0 h, (**b**) 1 h, (**c**) 2 h, and (**d**) 3 h.

Based on the BBR and DSR test results as well as taking into account the high-temperature deformation resistance and low-temperature performance of the asphalt binders in the previous sections, 0.35% was selected as the optimum RPCM content for morphological testing. A high-resolution fluorescence microscope (FM) was used for observing the microscopic phase of the RPCM dispersed in HMAB to evaluate the modification mechanism of the 0.35% RPCM. The fluorescent images of 0.35% RPCM-modified asphalt binder under different evolution (developing) times are presented in Figure 9.

Figure 9 visually shows distinctive changes in the morphology of asphalt binders with an increase in the evolution (developmental) time and it is clearly visible that no fluorescent reaction appears in the base asphalt binder area. The polymer material can display a fluorescent reaction under FM after it swells by absorbing light fractions in the asphalt binder matrix, while the asphalt binder phase cannot exhibit fluorescence [22]. With an increase in the evolution time, the difference between the RPCM and base asphalt binder becomes smaller and gradually form a uniform network distribution in the base asphalt binder. This means that RPCM has good compatibility characteristics with the base asphalt binder.

When the evolution time is further increased to 3 h, the continuous network structure is formed by RPCM while the asphalt binder phase transforms into a dispersed phase. This is mainly because the RPCM adsorbs the oil content from the base asphalt binder to form a continuous network structure of cross-distribution after swelling, which ultimately contributes to improving HMAB performance. However, the composition of RPCM is different from that of the base asphalt binder; thus, the RPCM is still a physical blend with the base asphalt blend, which cannot maximize the modification effects of RPCM.

## 5. Conclusions and Recommendations

This laboratory study was conducted to investigate the effects of different dosages of RPCM on the rheological properties and morphology of high-modulus asphalt binders. The base asphalt binder used comprised A-70# Petroleum asphalt binder with the RPCM dosages ranging from 0.25% to 0.45%, respectively. The following conclusions and recommendations were drawn:

- The temperature and frequency sweep test results showed that the addition of RPCM reduced the temperature sensitivity of the asphalt binder and correspondingly increased its stiffness (complex modulus). At dosages greater than 0.30%, RPCM exhibited superiority over SBS in enhancing the complex modulus of the asphalt binder.
- With the addition of RPCM under increasing temperature, the phase angle generally exhibited an increasing trend. This result suggested that modification with RPCM had the potential to improve the high-temperature elastic properties of the asphalt binder.
- The rutting parameter (G*/Sinδ) exhibited an increasing nonlinear trend with an increase in the RPCM dosage—illustrating the potential of RPCM modification to reduce the temperature sensitive of the asphalt binder and enhance its high-temperature rutting resistance potential. In comparison to SBS modification, RPCM exhibited superior performance for dosages greater than 0.30%, which was similar to the modulus results.
- The master curve graphical plots indicated that RPCM modification has more pronounced effects in the lower loading frequency domain corresponding to high temperatures when the viscoelastic asphalt binder is more prone to rutting. Based on the phase angle master curves, the SBS-modified asphalt binder tended to be more viscous and prone to high-temperature deformation than the RPCM-modified asphalt binders.
- With an increase in the RPCM content, the asphalt binder generally became softer (i.e., low *S*, low *λ*, and high *m value*, respectively), with better low-temperature crack resistance and decreased risk of cracking damage. However, the low-temperature performance of the asphalt binder significantly declined for RPCM contents greater than 0.35%—indicating that the optimum dosage should not exceed 0.35%.
- The morphological results from fluorescence microscopic imaging indicated that RPCM had good compactibility characteristics with A-70# Petroleum asphalt binder, and thus it could suitably be used as a modifier.

In general, RPCM exhibited satisfactory compactibility and potential for high-modulus asphalt binder modification to enhance the base asphalt binder’s rheological properties, both with respect to high- and low-temperature performance improvements. For the A-70# Petroleum asphalt binder that was evaluated, the optimum RPCM dosage was found to be 0.30~0.35%. While plausible results were obtained in this paper, future follow-up studies should include more base asphalt binders to further supplement and substantiate the findings reported herein, along with field correlations and validations. Overall, the study beneficially contributes to the literature through provision of a reference datum toward the exploratory usage of RPCM for high-modulus asphalt binder modification and performance enhancements.

## Figures and Tables

**Table 1:** Technical indices for AH-70 Petroleum asphalt binder.

Technical Indexes		Unit	Specification	Results
Penetration (25 °C, 100 g, 5 s)		0.1 mm	60~80	65
Penetration index, PI		—	−1.5~+1.0	−1.33
Softening point, T_R&B_		°C	≥46	50.5
Ductility (15 °C, 5 cm/min)		cm	≥100	138
Ductility (10 °C, 5 cm/min)		cm	≥15	19.3
Density @15 °C		g/cm^3^	/	1.02
Dynamic viscosity @60 °C		Pa∙s	≥180	210
Kinematic viscosity @135 °C		Pa.s	/	0.420
After RTFOT (163 °C 85 min)	Mass change	%	−0.8~+0.8	0.095
	Penetration ratio @25 °C	%	≥61	73
	Ductility (10°C, 5 cm/min)	cm	≥6	6.4
	Ductility (15 °C, 5 cm/min)	cm	/	50.0

**Table 2:** Technical indices of RPCM.

Size (mm)	Density (g/cm^3^)	State	Appearance	Mass-Flow Rate (g/10 min) (190 °C, 2.16 kg)
1~6	0.96	Solid	Black granular	≥2.0

## Data Availability

The data presented in this study are available on request from the corresponding author.

## References

[1] Zhu J., Birgisson B., Kringos N. (2014). Polymer modification of bitumen: Advances and challenges. Eur. Polym. J..

[2] Nanjegowda V.H., Biligiri K.P. (2020). Recyclability of rubber in asphalt roadway systems: A review of applied research and advancement in technology. Resour. Conserv. Recycl..

[3] Zaumanis M., Poulikakos L., Partl M. (2018). Performance-based design of asphalt mixtures and review of key parameters. Mater. Des..

[4] Xu T., Huang X. (2012). Investigation into causes of in-place rutting in asphalt pavement. Constr. Build. Mater..

[5] Walubita L.F., Fuentes L., Lee S.I., Dawd I., Mahmoud E. (2019). Comparative evaluation of five HMA rutting-related laboratory test methods relative to field performance data: DM, FN, RLPD, SPST, and HWTT. Constr. Build. Mater..

[6] Moghaddam T.B., Baaj H. (2020). The use of compressible packing model and modified asphalt binders in high-modulus asphalt mix design. Road Mater. Pavement Des..

[7] Chen Y., Wang H., Xu S., You Z. (2020). High modulus asphalt concrete: A state-of-the-art review. Constr. Build. Mater..

[8] Xiao F., Wang J., Yuan J., Liu Z., Ma D. (2020). Fatigue and Rutting Performance of Airfield SBS-Modified Binders Containing High Modulus and Antirutting Additives. J. Mater. Civ. Eng..

[9] Zhu H., Sun L., Yang J., Chen Z., Gu W. (2011). Developing Master Curves and Predicting Dynamic Modulus of Polymer-Modified Asphalt Mixtures. J. Mater. Civ. Eng..

[10] Geng H., Clopotel C.S., Bahia H.U. (2013). Effects of high modulus asphalt binders on performance of typical asphalt pavement structures. Constr. Build. Mater..

[11] Zou X., Sha A., Jiang W., Liu Z. (2017). Effects of modifier content on high-modulus asphalt mixture and prediction of fatigue property using Weibull theory. Road Mater. Pavement Des..

[12] Wang X., Qiu Y.-J., Xue S.-Y., Yang Y., Zheng Y. (2018). Study on durability of high-modulus asphalt mixture based on TLA and fibre composite modification technology. Int. J. Pavement Eng..

[13] Khiavi A.K., Naseri S. (2019). The effect of bitumen types on the performance of high-modulus asphalt mixtures. Pet. Sci. Technol..

[14] Xu X., Lu G., Yang J., Liu X. (2020). Mechanism and Rheological Properties of High-Modulus Asphalt. Adv. Mater. Sci. Eng..

[15] Zhu J., Ma T., Fan J., Fang Z., Chen T., Zhou Y. (2020). Experimental study of high modulus asphalt mixture containing reclaimed asphalt pavement. J. Clean. Prod..

[16] Zou X., Sha A., Jiang W., Huang X. (2015). Modification mechanism of high modulus asphalt binders and mixtures performance evaluation. Constr. Build. Mater..

[17] Wang C., Zhao L., Cao D. (2017). Experimental study on rheological characteristics and performance of high modulus asphalt binder with different modifiers. Constr. Build. Mater..

[18] JTG F40–2004 (2004). Technical Specification for Construction of Highway Asphalt Pavement.

[19] D’Angelo J.M., Fee F. (2020). “SUPERPAVE BINDER TESTS AND SPECIFICATIONS: HOW HAVE THEY PERFORMED IN THE REAL WORLD?,”, 2000, vol. 69. Assoc. Asph. Paving Technol. Proc..

[20] Bouldin M.G., Dongré R., D′angelo J. (2001). Proposed Refinement of Superpave High-Temperature Specification Parameter for Performance-Graded Binders. Transp. Res. Rec. J. Transp. Res. Board.

[21] ISO (2005). Plastics—Determination of the Melt Mass-Flow Rate (MFR) and the Melt Volume-Flow Rate (MVR) of Thermoplastics.

[22] Walubita L.F., Alvarez A.E., Simate G.S. (2011). Evaluating and comparing different methods and models for generating relaxation modulus master-curves for asphalt mixes. Constr. Build. Mater..

[23] Hu X., Fan S., Li X., Pan P., Fuentes L., Walubita L.F. (2020). Exploring the feasibility of using reclaimed paper-based asphalt felt waste as a modifier in asphalt-binders. Constr. Build. Mater..

[24] Bai T., Hu Z.-A., Hu X., Liu Y., Fuentes L., Walubita L.F. (2020). Rejuvenation of short-term aged asphalt-binder using waste engine oil. Can. J. Civ. Eng..

[25] ASTM (2016). ASTM D6373-16. Standard Specification for Performance Graded Asphalt Binder.

[26] Bahia H.U., Anderson D.A. (1995). Strategic highway research program binder rheological parameters: Background and comparison with conventional properties. Transp. Res. Rec..

[27] Williams M.L., Landel R.F., Ferry J.D. (1955). The Temperature Dependence of Relaxation Mechanisms in Amorphous Polymers and Other Glass-forming Liquids. J. Am. Chem. Soc..

